# The Group 3 Innate Lymphoid Cell Defect in Aryl Hydrocarbon Receptor Deficient Mice Is Associated with T Cell Hyperactivation during Intestinal Infection

**DOI:** 10.1371/journal.pone.0128335

**Published:** 2015-05-26

**Authors:** Sagie Wagage, Gretchen Harms Pritchard, Lucas Dawson, Elizabeth L. Buza, Gregory F. Sonnenberg, Christopher A. Hunter

**Affiliations:** 1 Department of Pathobiology, School of Veterinary Medicine, University of Pennsylvania, Philadelphia, Pennsylvania, United States of America; 2 Department of Medicine and Institute for Immunology, Perelman School of Medicine, Division of Gastroenterology, University of Pennsylvania, Philadelphia, Pennsylvania, United States of America; Charité, Campus Benjamin Franklin, GERMANY

## Abstract

Intestinal infection with the intracellular parasite *Toxoplasma gondii* results in the translocation of commensal bacteria to peripheral organs and the development of a T cell response specific to the microbiota. In naïve mice, the recently described RORγt^+^ group 3 innate lymphoid cell (ILC) population plays a critical role in promoting intestinal barrier function and limiting responses to gut-resident commensal bacteria. Given this role for group 3 ILCs, studies were performed to evaluate whether these cells might influence the immune response to mucosal infection with *T*. *gondii*. Phenotypic characterization of RORγt^+^ ILCs in *T*. *gondii* infected mice revealed that this population decreased following challenge but the population that remained expressed costimulatory molecules and IL-22. One factor that influences the maintenance of RORγt^+^ ILCs is the aryl hydrocarbon receptor (AHR), a ligand-activated transcription factor, and *Ahr^-/-^* mice have a marked defect in the lamina propria group 3 ILC population. When *Ahr^-/-^* mice were challenged with *T*. *gondii*, they lost more weight than wild type controls. This disease course in *Ahr^-/-^* animals was associated with increased T cell responses to *Toxoplasma* antigen and crude commensal antigen preparations. Together, these data suggest that group 3 ILCs have a role in limiting T cell activation during intestinal infection.

## Introduction

The ability of cells of the immune system to sense and react to environmental stimuli plays a crucial role in the maintenance of steady state conditions and in the effective control of infection while limiting immune-mediated damage to the host. Immune cells use a variety of sensors to respond to environmental cues, and the aryl hydrocarbon receptor (AHR), a ligand-activated transcription factor, provides these cells with one means to detect and respond to environmental signals [1, 2]. The AHR binds to structurally diverse agonists that include endogenous compounds such as certain tryptophan metabolites and synthetic molecules exemplified by 2,3,7,8-tetrachlorodibenzo-p-dioxin [3]. AHR ligands derived from plants can also be obtained through the diet [3], which makes the intestines a major site of exposure to AHR agonists. Accordingly, signaling through this transcription factor has multiple effects on intestinal immune responses. AHR activity in the intestine is required for the development of isolated lymphoid follicles and cryptopatches, and contributes to the maintenance of intraepithelial lymphocytes and group 3 innate lymphoid cells (ILCs) [4–7]. This RORγt^+^ ILC population has a critical role in the regulation of intestinal barrier function in naïve mice and IL-22 expression by these cells limits the dissemination of the microbiota to distal sites [8]. Group 3 ILCs are also able to present antigen but because they typically lack the expression of costimulatory molecules, they are thought to promote T cell tolerance to commensal bacteria [9].

The gastrointestinal microbiota influences normal host physiology as well as the immune response to infection with a variety of pathogenic microorganisms [10]. For example, oral infection with the intracellular parasite *Toxoplasma gondii* can lead to immune-mediated local tissue damage associated with the translocation of commensal bacteria and the development of a T cell response specific to the microbiota [11, 12]. This infection-induced dissemination of commensal bacteria is controlled by neutrophils, which form structures in the lumen that restrict contact between the microbiota and the intestinal epithelium [13].

Given the role for group 3 ILCs in limiting responses to commensal bacteria at the steady state, studies were performed to evaluate whether these cells might influence the immune response to mucosal infection with *T*. *gondii*. Following infection, there was a marked decrease in the frequency of RORγt^+^ ILCs, but the population that remained expressed costimulatory molecules and IL-22. In order to study the possible effects of RORγt^+^ ILCs during infection, *Ahr* ^*-/-*^ mice, which have a prominent defect in this population, were challenged with *T*. *gondii*. Infected *Ahr* ^*-/-*^ animals lost more weight than wild type controls and exhibited increased T cell responses when stimulated with *Toxoplasma* antigen and crude commensal antigen preparations. These results suggest that group 3 ILCs have a role in limiting T cell activation during infection.

## Materials and Methods

### Mice and infections

*Ahr* ^-/-^ mice that had been backcrossed onto a C57/Bl6 background for 21 generations were obtained from Dr. Christopher A. Bradfield (University of Wisconsin School of Medicine and Public Health, Madison, WI). C57/Bl6 mice were purchased from the Jackson Laboratory (Bar Harbor, ME). CBA/J mice were purchased from the National Cancer Institute Animal Production Program (Frederick, MD). All mice were bred and housed in specific pathogen-free facilities at the University of Pennsylvania in accordance with institutional guidelines, and these specific studies were approved by the University of Pennsylvania Institutional Animal Care and Use Committee (Protocol 805432). Mice were euthanized using CO_2_ in a custom flow metered chamber. For infections, Me49 cysts were harvested from the brains of chronically infected CBA/J mice and experimental animals were orally gavaged with 20 or 100 cysts. Infected mice were monitored daily and at the timepoints assessed in this study these animals did not show adverse effects that necessitated sacrifice or the use of analgesics. For the analysis of parasite burdens, DNA was isolated from tissues using the High Pure PCR Template Preparation kit (Roche, Indianapolis, IN). Parasite DNA levels were determined by RT-PCR as previously described [14].

### Cell isolation

Spleens and lymph nodes were dissociated through a 40μm filter. Red blood cells in the spleens were lysed with 0.86% ammonium chloride (Sigma) in sterile water. For cell isolation from the gut, small intestines were cut longitudinally after removing the Peyer’s patches and the contents were washed in RPMI. Epithelial cells were stripped with 5mM EDTA and 1mM DTT in RPMI, and the tissue was digested with 0.16U/mL Liberase TL (Roche) and 0.05% DNAse. The suspension was passed through a 70μm filter, and then passed through a 40μm filter.

### ELISAs

Cells were cultured at 1–2.5x10^6^ cells/ml in complete RPMI (10% heat inactivated FCS, 2mM glutamine, 10U/ml penicillin, 10μg/ml streptomycin, 1mM sodium pyruvate, 1% nonessential amino acids, 5x10^-5^M 2-ME) with 62μg/ml soluble *Toxoplasma* antigen. Supernatants were collected after 48 hours and cytokines were detected by ELISA. For IL-10 ELISAs, Immulon 4HBX plates (Thermo Fisher Scientific, Waltham, MA) were coated with anti-IL-10 (clone JES5-2A5) (BD Pharmingen San Diego, CA), blocked in 5% FBS in PBS, and loaded with samples. Biotinylated anti-IL-10 (clone JES5-16E3) was used for detection followed by peroxidase-conjugated streptavidin (Jackson ImmunoResearch Laboratories, West Grove, PA), SureBlue (KPL, Gaithersburg, MD) and TMB Stop Solution (KPL).

### Stimulation with antigen preparations

Crude commensal antigen preparations were generated as previously described [9]. Briefly, small intestinal contents were collected in PBS and centrifuged at 1000 rpm to eliminate large aggregates. The supernatant containing the majority of bacteria was collected and centrifuged at 8000 rpm to pellet the bacteria. The pellet was resuspended in PBS, sonicated on ice, centrifuged at 20000g for 10 minutes, and the supernatant was used as a source of bacterial antigens. To evaluate T cell IFN-γ production, splenocytes were stimulated with 10μg/mL of the commensal antigen preparations or with 10μg/mL soluble *Toxoplasma* antigen for 5 hours. Brefeldin A was then added to the cultures overnight. The cells were surface stained, fixed, and stained for IFN-γ.

### Flow cytometry

For intracellular cytokine staining, cells were incubated with brefeldin A (Sigma) and PMA and ionomycin for 4 hours, surface stained, and fixed with 4% paraformaldehyde (Electron Microscopy Sciences, Hatfield, PA). Cells were then permeabilized with 0.5% saponin (Sigma) and stained for cytokines. For surface staining, samples were washed in flow cytometry buffer containing 1% BSA (Sigma) and 2mM EDTA (Invitrogen) in PBS, Fc blocked with 2.4G2 and normal rat IgG (Invitrogen), and stained with monoclonal antibodies. For the detection of T-bet, RORγt, FoxP3, and Ki67, cells were surface stained and then stained intracellularly for these proteins using the FoxP3/transcription factor staining buffer set (eBioscience). The following antibodies were purchased from eBioscience: T-bet eFluor660, CD3 APC-eFluor780, TNF-α FITC, IFN-γ PeCy7, IFN-γ PE, FoxP3 eFluor450, RORγt PE, CD3 PerCP-Cy5.5, CD5 PerCP-Cy5.5, CD11c APC-eFluor780, B220 APC-eFluor780, CD80 FITC, MHC II APC. The following antibodies were purchased from BD Pharmingen: Ki67 FITC, CD4 PE, and CD8 PerCP-Cy5.5. The following antibodies were purchased from BioLegend: CXCR3 PeCy7, NK1.1 PerCP-Cy5.5, CD90.2 AF700, IL-22 Alexa Fluor 647. Live/Dead Fixable Aqua dead cell stain (Life Technologies) was used to discriminate between live and dead cells. Samples were run on a FACSCanto II or LSRFortessa and data was analyzed using Flowjo software (Tree Star, Inc., Ashland, OR).

### Statistical analysis

Statistical significance was determined using Student’s t tests, which were performed using Prism software (GraphPad software, Inc. La Jolla, CA). P values of less than 0.05 were considered significant. Error bars in all graphs represent the standard deviation.

## Results

**Fig 1:**
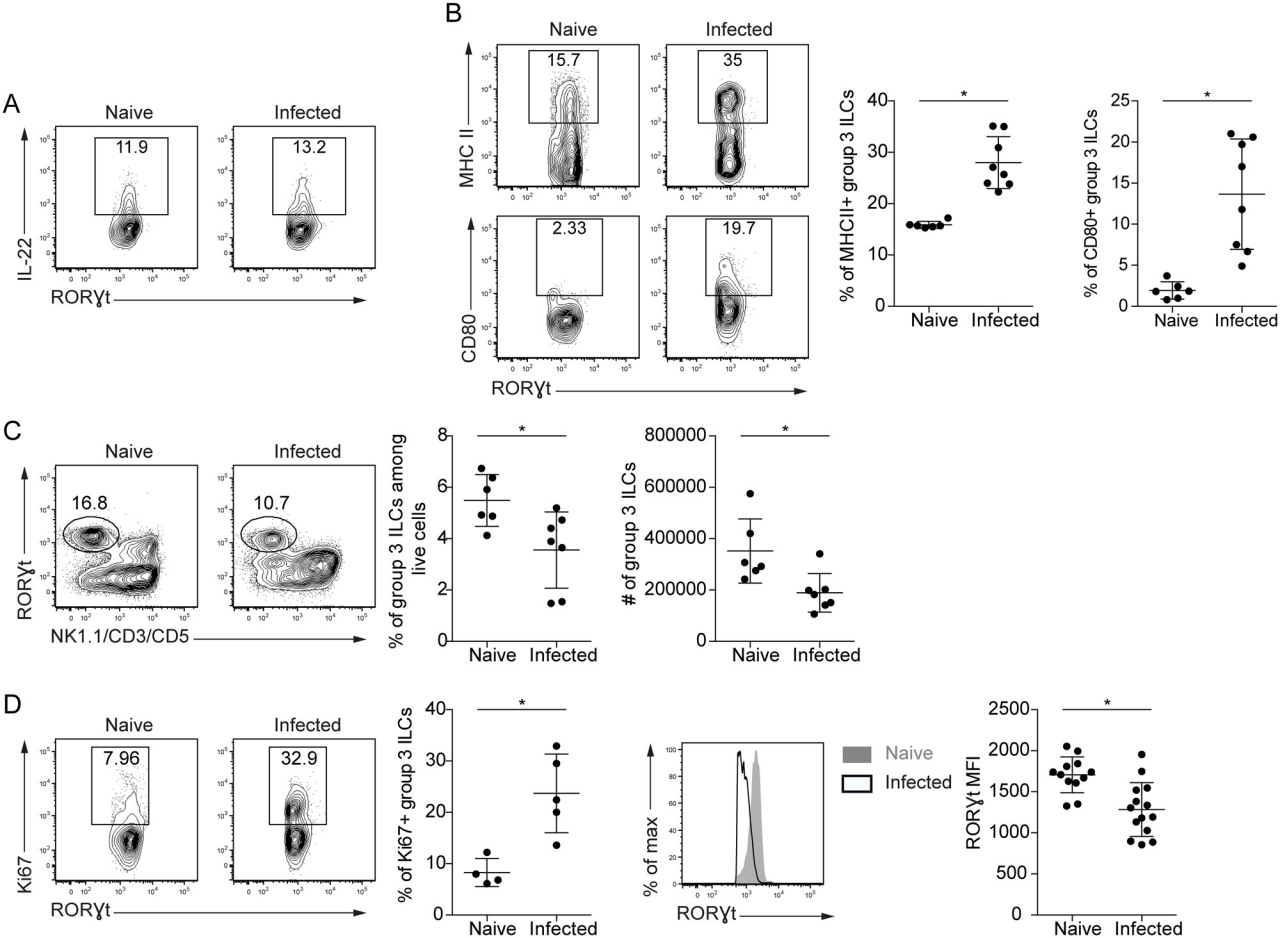
Phenotype of group 3 ILCs during toxoplasmosis. Wild type mice were infected orally with 20 Me49 cysts for 9 days, and cells were isolated from the lamina propria of infected mice or naïve controls. Group 3 ILCs were gated as live NK1.1^-^CD3^-^CD5^-^CD11c^-^B220^-^CD90.2^+^RORγt^+^ cells. Results are pooled from 2–5 separate experiments. **(A)** IL-22 production by group 3 ILCs following stimulation with PMA/ionomycin. **(B)** MHC II and CD80 expression by group 3 ILCs. **(C)** Frequency and number of group 3 ILCs in the lamina propria. The plots on the left are gated on live CD90.2^+^CD11c^-^B220^-^ cells. **(D)** RORγt and Ki67 expression by group 3 ILCs.

To evaluate a possible role for group 3 ILCs during *T*. *gondii* infection, experiments were performed to assess the phenotype of these cells following oral challenge with this parasite. Group 3 ILCs in naive mice expressed IL-22, and this was not significantly altered during toxoplasmosis (Fig 1A). Since RORγt^+^ ILCs are able to present antigen [9], their expression of MHC class II and the costimulatory molecule CD80 was evaluated. An increased frequency of group 3 ILCs expressed MHC class II during toxoplasmosis (Fig 1B). In naïve mice, CD80 was not widely expressed by RORγt^+^ ILCs, but after infection this costimulatory molecule was expressed by a subset of group 3 ILCs (Fig 1B). Notably, oral infection with *T*. *gondii* led to a decrease in the frequency and number of RORγt^+^ ILCs in the lamina propria (Fig 1C), which was associated with decreased expression of RORγt by these cells in infected mice and increased expression of Ki67 (Fig 1D). These results show that challenge with *T*. *gondii* led to marked changes in the group 3 ILC population, and suggest that despite the overall reduction in this population, there was an increased frequency of these cells undergoing proliferation.

**Fig 2:**
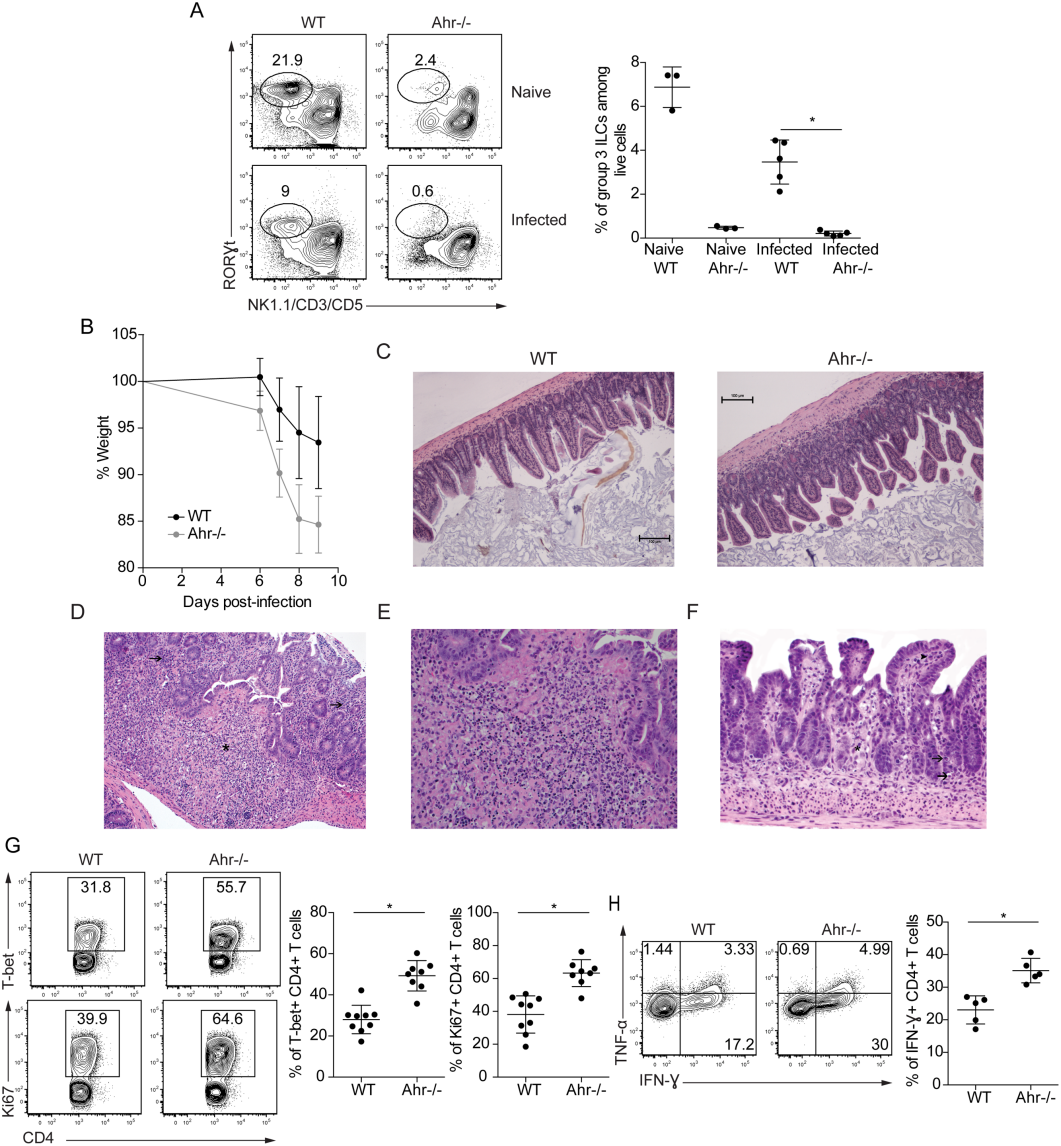
*Ahr* ^*-/-*^ mice exhibit increased T cell activation following infection. Wild type or *Ahr* ^*-/-*^ mice were orally infected with *T*. *gondii* for 9 days. **(A)** Group 3 ILC frequency in the lamina propria of wild type or *Ahr* ^*-/-*^ mice following infection. The plots on the left are gated on live CD90.2^+^CD11c^-^B220^-^ cells. (**B)** Weight loss was monitored at various days post-infection. Data are pooled from 2 experiments with 6–8 mice per group. **(C)** H&E staining of small intestinal tissue sections. **(D)** H&E staining of small intestinal tissue from an infected *Ahr* ^*-/-*^ mouse. The Peyer’s patch exhibits severe lymphocytolysis (*) and the lamina propria of adjacent villi is expanded by primarily lymphocytes and plasma cells (➜). **(E)** A higher magnification image of the section in Fig 2C shows that the Peyer’s patch exhibits severe lymphocytolysis characterized by pyknosis and other cellular debris. **(F)** H&E staining of small intestinal tissue from an infected *Ahr* ^*-/-*^ mouse shows crypt loss (*) and multifocal necrotic enterocytes (➜). The lamina propria of the villi is expanded by lymphocytes and plasma cells (❋). **(G)** Expression of T-bet and Ki67 by FoxP3^-^ CD4^+^ T cells in the mesenteric lymph nodes of infected mice. Results are pooled from 3 separate experiments. **(H)** Cytokine production by CD4^+^ T cells following stimulation with PMA/ionomycin. Data are pooled from 2 experiments.

The infection-induced changes in the RORγt^+^ ILC population raised the question of whether these cells influence the immune response to toxoplasmosis. Notably, naïve *Ahr* ^*-/-*^ mice have a marked reduction in group 3 ILCs in the lamina propria [5–7], and therefore experiments were performed to evaluate the responses of these mice to *T*. *gondii*. The defect in the RORγt^+^ ILC population seen in naïve mice in the absence of the AHR was also apparent in infected *Ahr* ^*-/-*^ animals (Fig 2A). Following oral challenge, weight loss in wild type and *Ahr* ^*-/-*^ mice was monitored to determine disease progression, which indicated that infected *Ahr* ^*-/-*^ mice lost more weight than wild type animals (Fig 2B). Histological evaluation of small intestinal tissue from multiple experiments with a total of 16 wild type and 12 *Ahr* ^*-/-*^ mice that had been infected for 9 days indicated that all infected animals had lymphoplasmacytic inflammation expanding the lamina propria of the small intestine with areas of enterocyte loss and necrosis. The Peyer’s patches exhibited mild to severe lymphocytolysis (Fig 2C–2F). Grading of histological sections of the small intestine by an anatomical pathologist revealed that wild type mice typically had mild to moderate inflammation whereas *Ahr* ^*-/-*^ animals had moderate to severe disease, consistent with the increased weight loss observed in these mice.

Since the development of a Th1 CD4^+^ T cell response contributes to immune-mediated pathology during oral toxoplasmosis [15], studies were performed to assess whether the absence of the AHR influenced T cell responses. In naive wild type and *Ahr* ^*-/-*^ animals, similar frequencies of CD4^+^ T cells expressed Ki67 and the Th1 associated transcription factor T-bet (data not shown). However, compared to wild type mice, the CD4^+^ T cell population in infected *Ahr* ^*-/-*^ animals expressed higher levels of T-bet and elevated amounts of Ki67 (Fig 2G). Consistent with the increased activation phenotype of *Ahr* ^*-/-*^ CD4^+^ T cells during infection, these lymphocytes produced increased amounts of IFN-γ following stimulation with PMA/ionomycin (Fig 2H). Collectively, these results indicated that *Ahr* ^*-/-*^ mice developed a hyperactivated CD4^+^ T cell response during toxoplasmosis and were more susceptible to this challenge.

**Fig 3:**
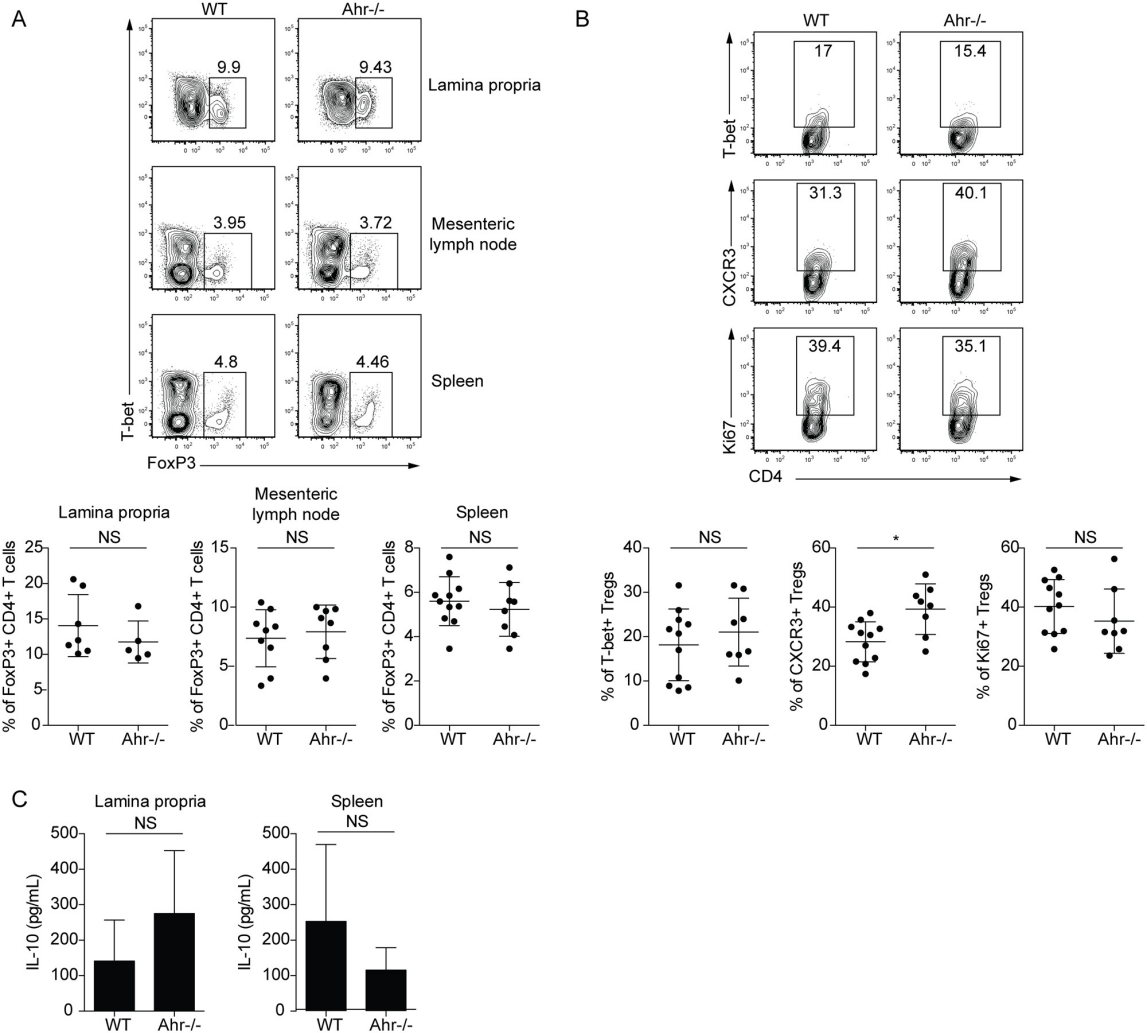
Treg phenotype and IL-10 production in orally infected *Ahr* ^*-/-*^ mice. *Ahr* ^*-/-*^ mice or wild type controls were infected orally with 20 Me49 cysts for 9 days. Results are pooled from 2–3 separate experiments. **(A)** Frequency of Tregs in the indicated tissues of wild type or *Ahr* ^*-/-*^ mice. In the lamina propria, the plots shown are gated on live CD45^+^CD3^+^CD4^+^ cells, and Tregs were gated as live CD45^+^CD3^+^CD4^+^FoxP3^+^ cells. For the mesenteric lymph node and spleen, the plots shown are gated on CD3^+^CD4^+^ cells, and Tregs were gated as CD3^+^CD4^+^FoxP3^+^ cells. **(B)** Expression of T-bet, CXCR3, and Ki67 on Tregs in the spleens of wild type or *Ahr* ^*-/-*^ mice. The plots are gated on CD3^+^CD4^+^FoxP3^+^ cells. **(C)** IL-10 secretion by cells isolated from the lamina propria or the spleen following restimulation with soluble *Toxoplasma* antigen. Results are pooled from 2 separate experiments with a total of 5–7 mice per group.

Since AHR signaling can promote Treg cell development [16, 17] and the production of IL-10 [18–21], it was possible that alterations in these pathways would contribute to the increased T cell activation seen in *Ahr* ^*-/-*^ mice. However, it should be noted that Treg frequencies were similar in infected wild type and *Ahr* ^*-/-*^ animals (Fig 3A). In addition, Treg expression of T-bet and CXCR3, which promote Treg function by allowing these cells to migrate to sites of inflammation and limit Th1 responses [22, 23], was not impaired in the spleen, mesenteric lymph nodes, or lamina propria of *Ahr* ^*-/-*^ animals (Fig 3B and data not shown). Similarly, no major defect in IL-10 production following restimulation with *Toxoplasma* antigen was detected in infected *Ahr* ^*-/-*^ mice (Fig 3C). These results indicate that the increased CD4^+^ T cell activation seen in these animals was not mediated by overt defects in the Treg cell population or the production of IL-10.

Since infected *Ahr* ^*-/-*^ mice have a marked reduction in group 3 ILCs in the lamina propria and RORγt^+^ ILCs promote tolerance to the microbiota [9], experiments were performed to assess whether the hyperactive T cell response in *Ahr* ^*-/-*^ animals was related to increased responses to commensals during infection. Splenocytes from infected mice were stimulated with parasite antigen or with commensal antigen preparations generated from small intestinal luminal contents, and T cell production of IFN-γ production was assessed. Since the composition of the microbiota changes markedly following infection with *T*. *gondii* [13, 24], crude commensal antigen preparations were made from the luminal contents of naïve and infected animals. However, it was possible that the commensal antigen preparation generated from infected animals contained a low level of *T*. *gondii*, since the parasite can be found in the intestinal lumen following infection [25]. To evaluate this possibility, mice were immunized intraperitoneally with a replication deficient strain of *T*. *gondii*, which would not lead to disruption of the intestinal barrier or the generation of a microbiota-specific T cell response. Splenocytes from these mice were stimulated with different antigen preparations, and IFN-γ production was analyzed. Stimulation with *Toxoplasma* antigen induced IFN-γ expression by these cells, but stimulation with the commensal antigen preparation from infected mice elicited negligible levels of IFN-γ compared to cells that were unstimulated or treated with commensal antigen from naïve mice (data not shown). These results indicated that the commensal antigen generated from infected mice did not contain sufficient levels of parasite protein to induce a large *T*. *gondii*-specific response.

**Fig 4:**
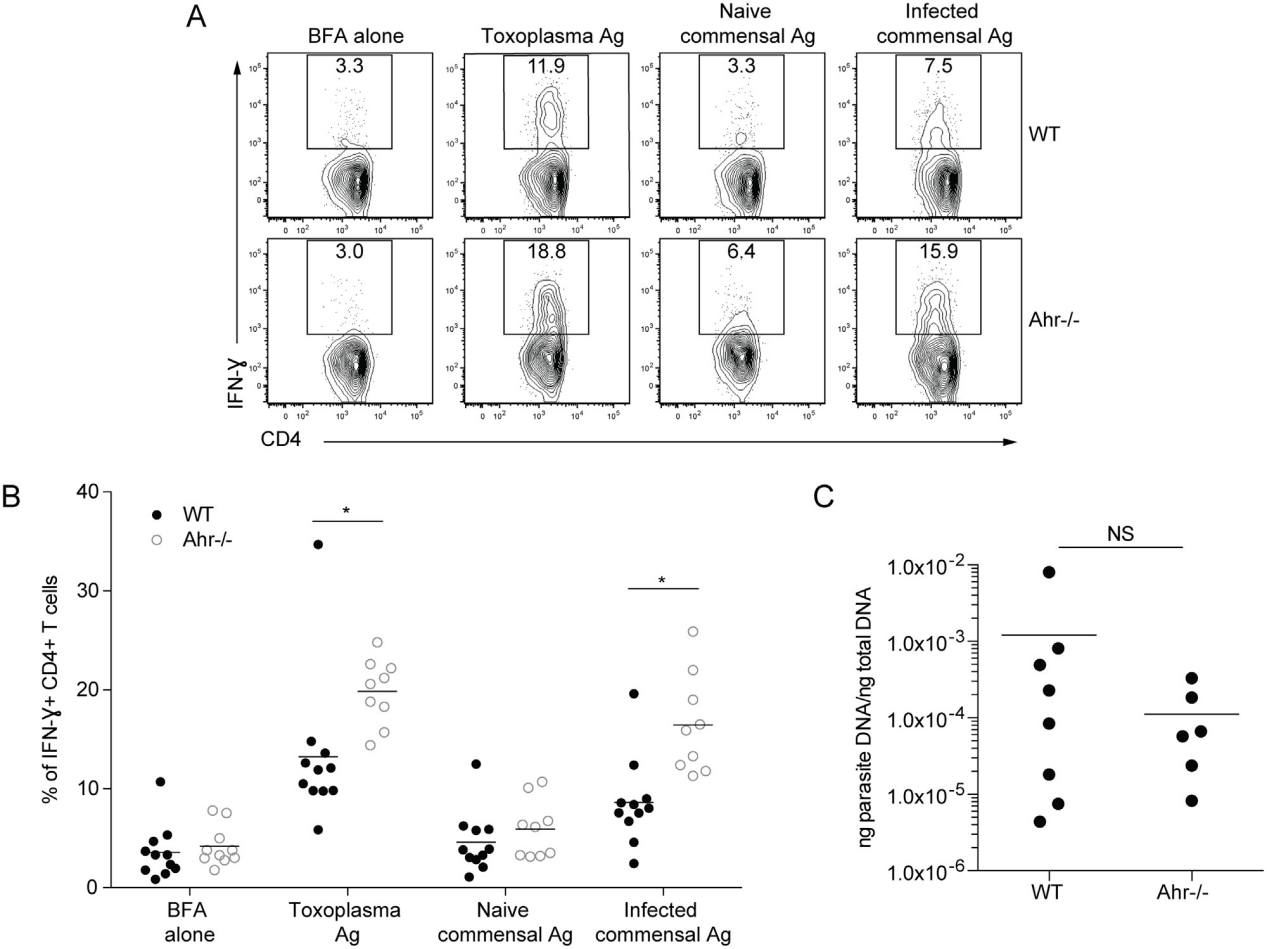
Increased T cell responses to *Toxoplasma* antigen and crude commensal antigen in *Ahr* ^*-/-*^ mice following infection. Wild type or *Ahr* ^*-/-*^ mice were orally infected with 100 *T*. *gondii* cysts for 7 days. **(A, B)** Splenocytes were stimulated with the indicated antigen preparations for 5 hours and then incubated overnight with brefeldin A. The cells were stained to assay IFN-γ expression by CD4^+^ T cells. The graph shows pooled data from 3 independent experiments. **(C)** Parasite burdens in the terminal ileum were assayed by RT-PCR. Results are pooled from 2 separate experiments.

To evaluate the responses to the commensal antigen in wild type and *Ahr* ^*-/-*^ mice, splenocytes from mice that had been orally infected with the Me49 strain of *T*. *gondii* were stimulated. Interestingly, stimulation of splenocytes from infected wild type mice with crude commensal antigen generated from naïve mice induced a low level of IFN-γ production, while stimulation with commensal antigen from infected mice elicited a higher frequency of IFN-γ ^+^ CD4^+^ T cells (Fig 4A and 4B). When splenocytes from *Ahr* ^*-/-*^ mice were stimulated with commensal antigen from infected mice, there was an increased percentage of CD4^+^ T cells that produced IFN-γ (Fig 4A and 4B). An increased frequency of CD4^+^ T cells from *Ahr* ^*-/-*^ animals also expressed IFN-γ upon stimulation with *Toxoplasma* antigen (Fig 4A and 4B). At this timepoint, parasite burdens were similar between infected wild type and *Ahr* ^*-/-*^ animals (Fig 4C). It should be noted that more significant differences in T cell IFN-γ expression from wild type and *Ahr* ^*-/-*^ mice were observed when infecting with a higher dose of 100 *T*. *gondii* cysts (data not shown), consistent with the notion that increased parasite doses induce more severe intestinal damage [26, 27]. Together, these results suggest that increased T cell responses to commensal antigen contributed to the T cell hyperactivation seen in *Ahr* ^*-/-*^ mice.

## Discussion

Oral challenge of wild type mice with *T*. *gondii* led to a reduction in the frequency and number of group 3 ILCs in the lamina propria as well as decreased RORγt expression by these cells. Previous adoptive transfer experiments have found that a subset of group 3 ILCs can downregulate RORγt expression and develop the ability to produce IFN-γ, a process that is promoted by IL-12 signals [28]. IL-12 expression during oral toxoplasmosis may induce a similar conversion of RORγt^+^ ILCs into IFN-γ expressing ILCs, which were recently shown to have a protective role in the context of this infection [29]. Notably, downregulation of RORγt in group 3 ILCs is associated with the proliferation of these cells [28], and an increased frequency of group 3 ILCs expressed Ki67 following challenge with *T*. *gondii*. Therefore, the reduction in the group 3 ILC population during infection may be due a conversion of these cells into IFN-γ producing ILCs. Alternatively, the decrease in the RORγt^+^ ILC population in spite of its increase in Ki67 expression may be due to an increased level of apoptosis in these cells. Given the role of RORγt^+^ ILCs in promoting the anatomical containment of commensal bacteria [8], a reduction in this cell population could contribute to the barrier dysfunction that occurs during toxoplasmosis [11, 12]. Since RORγt^+^ ILCs promote tolerance to commensal bacteria in naïve mice [9], decreases in this cell population might facilitate the induction of immune responses to infection. This effect would be analogous to the suggestion that the Treg “crash” seen during acute toxoplasmosis facilitates the emergence of an effective T cell response [30].

Although the RORγt^+^ ILC population was markedly reduced during toxoplasmosis, some of the cells that remained at 9 days post-infection expressed MHC II and CD80. The increased frequency of MHC II expressing RORγt^+^ ILCs seen after challenge may be due to a selective enrichment of this subset of cells, as MHC II is expressed by a sub-population of group 3 ILCs in naïve mice [9]. Alternatively, a recent study found that stimulation with IL-1β or Toll-like receptor (TLR) ligands can promote the expression of costimulatory molecules by RORγt^+^ ILCs [31]. Since IL-1β is expressed in the intestine following *T*. *gondii* infection [32], and there are TLR ligands produced by the parasite [33] or by commensal bacteria [12], it is possible that these signals contribute to the group 3 ILC CD80 expression seen during toxoplasmosis. Regardless, additional studies are needed to understand how CD80 expression impacts RORγt^+^ ILC function during infection.

Although the AHR can contribute to Treg development [17] and T cell IL-10 production [20], processes that constrain the immune response during toxoplasmosis [34, 35], no deficiencies in these pathways were observed in infected *Ahr* ^*-/-*^ animals. Notably, FoxP3^-^ T-bet^+^ Th1 cells are thought to be the critical source of IL-10 during toxoplasmosis [36], but since previous studies have shown that the AHR is not expressed at detectable levels in Th1 cells it was unlikely that AHR activity would directly affect IL-10 production by this population [37]. The group 3 ILC defects seen in *Ahr* ^*-/-*^ mice raised the possibility that this phenotype could contribute to the increased T cell activation seen in AHR deficient animals following infection. Consistent with a role for these innate cells in limiting microbiota-specific responses during toxoplasmosis, an increased frequency of CD4^+^ T cells in *Ahr* ^*-/-*^ mice produced IFN-γ following stimulation with crude commensal antigen preparations. However, it is important to note that the AHR affects multiple aspects of the immune response, and other AHR functions may contribute to the phenotype of *Ahr* ^*-/-*^ mice during *T*. *gondii* infection. For example, AHR activity also affects the function of macrophages and dendritic cells [18, 19].

Since IL-22 has been shown to promote tissue pathology during toxoplasmosis [38–40], it was unexpected that *Ahr* ^*-/-*^ mice with a marked defect in IL-22-producing RORγt^+^ ILCs would develop increased intestinal pathology. These phenotypes may be related to differences in the microbiomes of *Ahr* ^*-/-*^ and IL-22 deficient mice. IL-22 deficient animals exhibit an altered microbiome compared to wild type mice [41], but the composition of the microbiota in wild type and *Ahr* ^*-/-*^ mice is comparable [5]. Alternatively, these phenotypic differences may be due to additional effects of the AHR in immunity beyond IL-22 induction. For example, functions of RORγt^+^ ILCs other than their IL-22 expression, such as antigen presentation, may influence the phenotype of infected *Ahr* ^*-/-*^ animals.

A previous report in which *Ahr* ^*-/-*^ mice were infected intraperitoneally with *T*. *gondii* found that these mice were more susceptible during chronic infection, which was associated with decreased levels of serum IL-10 and decreased parasite burdens in the brain [42]. In contrast, following oral infection, there were no overt defects in IL-10 expression and wild type and *Ahr* ^*-/-*^ animals had similar parasite burdens during the acute stage. Together, these results suggest that the AHR has context-dependent effects following different routes of challenge and/or in acute and chronic infection. One potential explanation for these different roles is that relevant sources of AHR ligands are likely to vary in distinct settings and can have disparate immunological effects [17]. In the gut, the ability of dietary compounds to activate the AHR contributes to the maintenance of group 3 ILCs [6], while other AHR agonists can be produced by commensal bacteria [43]. It is unclear whether the infection-induced perturbations to the gut microbiota that occur during toxoplasmosis influence AHR activity [13, 24], but *T*. *gondii* can also produce the AHR agonist lipoxin A_4_ [44, 45], and the AHR has been implicated in the direct sensing of pathogen-associated molecular patterns [46]. In addition to these exogenous sources of AHR ligands, compounds that activate this transcription factor can be produced as part of the host immune response to the parasite. For example, L-kynurenine, which is generated by tryptophan degradation catalyzed by the IFN-γ induced enzyme indoleamine 2,3 dioxygenase, is found in multiple tissues during toxoplasmosis and acts as an AHR agonist [3, 47]. Thus, sensing through the AHR of environmental, pathogen, or host-derived compounds could potentially contribute to its effects during oral infection with *T*. *gondii*.
